# The microbiome of a perennial cereal differs from annual winter wheat only in the root endosphere

**DOI:** 10.1093/ismeco/ycae165

**Published:** 2024-12-23

**Authors:** Kristina Michl, Makoto Kanasugi, Alena Förster, Regina Wuggenig, Sulemana Issifu, Katarzyna Hrynkiewicz, Christoph Emmerling, Christophe David, Benjamin Dumont, Linda-Maria Dimitrova Mårtensson, Frank Rasche, Gabriele Berg, Tomislav Cernava

**Affiliations:** Institute of Environmental Biotechnology, Graz University of Technology, Graz 8010, Austria; Department of Microbiology, Faculty of Biological and Veterinary Sciences, Nicolaus Copernicus University, Torun 87-100, Poland; Leibniz Centre for Agricultural Landscape Research (ZALF), Müncheberg 15374, Germany; Department of Soil Science, Faculty of Regional and Environmental Sciences, University of Trier, Trier 54286, Germany; Institute of Environmental Biotechnology, Graz University of Technology, Graz 8010, Austria; Department of Agronomy in the Tropics and Subtropics, Institute of Agricultural Sciences in the Tropics (Hans-Ruthenberg-Institute), University of Hohenheim, Stuttgart 70593, Germany; Department of Microbiology, Faculty of Biological and Veterinary Sciences, Nicolaus Copernicus University, Torun 87-100, Poland; Leibniz Centre for Agricultural Landscape Research (ZALF), Müncheberg 15374, Germany; Department of Soil Science, Faculty of Regional and Environmental Sciences, University of Trier, Trier 54286, Germany; Agroecology and Environment Research Unit, ISARA, Lyon 69364, France; University of Liege - Gembloux Agro-Bio Tech, Plant Sciences Axis, Crop Science lab., Gembloux 5030, Belgium; Department of Biosystems and Technology, Swedish University of Agricultural Sciences, Lomma 23422, Sweden; Department of Agronomy in the Tropics and Subtropics, Institute of Agricultural Sciences in the Tropics (Hans-Ruthenberg-Institute), University of Hohenheim, Stuttgart 70593, Germany; International Institute of Tropical Agriculture, P.O. Box 30772-00100, Nairobi, Kenya; Institute of Environmental Biotechnology, Graz University of Technology, Graz 8010, Austria; Leibniz-Institute for Agricultural Engineering and Bioeconomy Potsdam, Potsdam 14469, Germany; Institute for Biochemistry and Biology, University of Potsdam, Potsdam 14476, Germany; Institute of Environmental Biotechnology, Graz University of Technology, Graz 8010, Austria; School of Biological Sciences, Faculty of Environmental and Life Sciences, University of Southampton, Southampton SO171BJ, United Kingdom

**Keywords:** perennial grain, plant microbiome, rhizosphere, root endophytes, amplicon sequencing

## Abstract

The intensification of agriculture has led to environmental degradation, including the loss of biodiversity. This has prompted interest in perennial grain cropping systems to address and mitigate some of these negative impacts. In order to determine if perennial grain cultivation promotes a higher microbial diversity, we assessed the endophytic microbiota of a perennial grain crop (intermediate wheatgrass, *Thinopyrum intermedium* L.) in comparison to its annual counterpart, wheat (*Triticum aestivum* L.). The study covered three sampling sites in a pan-European gradient (Sweden, Belgium, and France), two plant genotypes, three plant compartments (roots, stems, and leaves), and two sampling time points. We observed that the host genotype effect was mainly evident in the belowground compartment, and only to a lesser extent in the aboveground tissues, with a similar pattern at all three sampling sites. Moreover, intermediate wheatgrass roots harbored a different bacterial community composition and higher diversity and richness compared to their annual counterparts. The root bacterial diversity was influenced by not only several soil chemical parameters, such as the carbon:nitrogen ratio, but also soil microbial parameters, such as soil respiration and dehydrogenase activity. Consistent findings across time and space suggest stable mechanisms in microbiota assembly associated with perennial grain cropping, underscoring their potential role in supporting biodiversity within sustainable agricultural systems.

## Introduction

The ongoing intensification of agricultural practices has resulted in environmental changes and challenges, including the degradation of soil fertility and depletion of biodiversity [1, 2]. One proposed solution to address these negative consequences is the implementation of perennial grain cropping systems with deep-rooted plants, which is inspired by natural ecosystems [3]. One of the most promising varieties of perennial grain crops is intermediate wheatgrass [*Thinopyrum intermedium* (host) Barkworth & D.R. Dewey; trademarked as Kernza®] [4]. Perennial grain cropping offers a more sustainable approach for plant production and could help to reduce negative impacts of agriculture, as plants remain in the same field for multiple years and thereby provide a permanent soil cover [5]. However, further research is required to determine if perennial plants can retain specific ecosystem services under agricultural settings, such as maintenance of enhanced biodiversity [6].

Intermediate wheatgrass offers various ecosystem services, particularly connected to soil health [7]. Soil microorganisms are key for governing soil health and are one of the main sources from which plants select their endophytic microbiome [8, 9]. Land-use intensity can influence microbial community structures in soils. Perennial systems were shown to have distinct communities of soil earthworms, nematodes, protists, and bacteria [10–13]. Furthermore, perennial plants have been linked to higher microbial diversity and biomass in bulk and rhizosphere soil, which may be attributed to increased root exudation [14–16]. The root-associated microbiome of intermediate wheatgrass is not only distinct from surrounding bulk soil [17], but there are also observable differences to other deep-rooted plant species. Endophytic microorganisms inhabit the inner tissues of plants and can support the host plant during germination [18, 19], nutrient acquisition [20, 21], protect against diseases [22], and can confer abiotic stress tolerance [19, 23]. The plant microbiome is influenced by multiple drivers, including abiotic and biotic factors [24, 25]. Furthermore, the host plant genotype, compartment niche, and developmental stage are significant determinants of microbial assembly, processes by which species from a regional pool colonize and interact to form stable local communities [26–29]. Another critical aspect is the evolutionary history of plants, which correlates with the microbial communities associated with them [30]. Furthermore, domestication and breeding for high yield cultivars shaped the microbiota of our modern crops [31]. Recently, the loss of microbial diversity and specificity as well as potential beneficial associations in modern crop plants have been increasingly recognized, which highlights the significance of studying native ecosystems as a source of plant-beneficial endophytes [32]. Moreover, wild plants are more adept at forming beneficial interactions, while modern crops may be impacted in this ability [31].

We hypothesized that: (i) intermediate wheatgrass has a distinct bacterial composition and greater microbial diversity across compartments in comparison to annual wheat; (ii) in the case of the root microbiome, this diversity will be influenced by soil chemical and biological characteristics; and (iii) the root microbiome of intermediate wheatgrass will be less variable and more connected across time due to reduced environmental disturbances. To test these hypotheses, our objectives were to compare the bacterial endophyte communities across different plant compartments (roots, stems, leaves) at multiple sites (Sweden, Belgium, and France) and time points (2021 and 2022), focusing on how plant genotype, life cycle, and soil parameters influence microbial diversity and community assembly.

## Materials and methods

### Sample collection and study sites

Samples of intermediate wheatgrass [*T. intermedium* (host) Barkworth & D.R. Dewey; trademarked Kernza®] and winter wheat (*Triticum aestivum* L.) roots, stems, and leaves were collected in June 2021 in Sweden (55°40′8″N, 13°7′0″E), Belgium (50°33′36″N, 4°42′0″E), and France (45°39′11″N, 5°14′38″E). Analogous sampling was conducted in April 2022, except for root samples in Belgium, which were sampled in May 2022. More detailed information on the wheat cultivars and sampling sites can be found in Supplementary Data Table S1.

In total, 720 destructive samples were collected: 20 biological replicates (5 per 4 subplots) × 3 compartments (roots, stems, and leaves) × 2 genotypes (intermediate wheatgrass and winter wheat) × 3 field sites (Sweden, Belgium, and France) × 2 sampling time points (June 2021 and April 2022). Roots were collected with a split tube sampler (Royal Eijkelkamp, Giesbeek, Netherlands; diameter: 5.3 cm) at a depth of 5–15 cm. Stem and leaf samples were collected beforehand above the soil core sample. Since perennial wheatgrass can spread through rhizomes, sampling of individual plants was not possible and several plants were pooled into one biological replicate.

All plant samples were put in sterile bags, stored cooled, and sent within 48 h for further sample processing either to the Nicolaus Copernicus University (Torun, Poland) or Graz University of Technology (Graz, Austria; Table S1).

### Surface sterilization, deoxyribonucleic acid extraction, and 16S ribosomal ribonucleic acid gene fragment sequencing

Roots (pre-washed and separated from soil), leaves, and stems were weighed and sterilized with 70% EtOH for 1 min, followed by washing with sterile H_2_O for 1 min. Afterwards, the roots and aboveground plant samples were sterilized with 7.5% H_2_O_2_ for 6 or 4 min, respectively, and finally washed 5 times with sterile H_2_O. The surface sterilized plant material was stored at −20°C until further use.

The plant material (approximately 50 mg of roots, 100 mg of leaves, and 200 mg of stems) was disrupted using mortar and pestle and liquid nitrogen. Subsequently, total genomic DNA was extracted following the manufacturer’s instructions of the DNeasy PowerSoil Kit (Qiagen, Valencia, CA, USA). The samples were stored at −20°C until further use. For amplification of the V4 region of the 16S ribosomal ribonucleic acid (rRNA) gene fragment, the universal barcoded primers 515f- 806r (515f: 5′-GTGYCAGCMGCCGCGGTAA-3′; 806r: 5′-GGACTACNVGGGTWTCTAAT-3′) were used [33]. Peptide nucleic acid clamps (PNA) were included in the polymerase chain reaction (PCR) mix to interfere with the amplification of host plastid and mitochondrial 16S rRNA genes [34]. PCRs were carried out in 25 μl volumes and two technical replicates using the 2× KAPA Taq Ready Mix (Kapa Biosystems, USA), 1.5 μM PNA mix, 0.2 mM of each primer, PCR-grade water, and 1 μl undiluted template DNA. The cycling conditions were as follows: 96°C for 3 min, 30 cycles of 95°C for 30 s, 78°C for 5 s, 54°C for 30 s, 72°C for 20 s, and a final extension at 72°C for 30 s. Out of the 720 samples, 16 could not be amplified (Table S1). Technical replicates were pooled and combined (Table S1) in equimolar concentrations. The amplicon libraries were purified using the Wizard SV Gel and PCR Clean-Up System (Promega, Madison, WI, USA) before being sent to the sequencing provider Novogene (Cambridge, UK) for library preparation. Sequencing was done on an Illumina NovaSeq 6000 platform (2 × 250 bp paired-end reads).

### Characterization of soil chemical and biological parameters

Soil gravimetric water content was measured after drying sieved soil at 105°C for 24 h. Soil pH was determined with a pH Cond 340i glass electrode (WTW Ltd, Germany) using air-dried soil in a 0.01 M CaCl_2_ solution. Total soil organic carbon and total nitrogen were quantified using the Elemental Analyser vario EL cube (Elementar Ltd, Germany). Plant available phosphorus (P) and potassium (K) were extracted in a Ca-acetate-lactate (CAL) solution according to Schüller [35]. Quantification of P was based on the colorimetric method of Murphey et al. [36] and measured using a photometer (UV-1650 PC; Shimadzu Europe GmbH, Duisburg, Germany). Determination of K was done using a flame atomic absorption spectroscopy (AA240 FS, Varian GmbH, Darmstadt, Germany).

Soil microbial carbon and nitrogen were determined with moist soil (adjusted to approx. 50% of maximum water holding capacity) according to the chloroform fumigation extraction method [37]. Extracts were analyzed with a TOC-TN Analyzer (Shimadzu TOC-V + TNN, Kyoto, Japan). Soil microbial respiration was determined according to Heinemeyer *et al.* [38] with moist soil samples. Released CO_2_ was assessed automatically by an infrared gas analyzer (ADC Model 225-MK3, Hoddesdon, England). Dehydrogenase activity (DHA) was determined based on a method presented by Thalmann [39]. Moist soil samples were incubated with a triphenyl tetrazolium chloride (TTC) solution dissolved in 0.1 molar Tris buffer for 24 h at 27°C. After 2 h of reaction time with shaking at regular intervals, the colored sample was filtrated and the liquid phase was measured at 546 nm against blank values on a spectrometer (Shimadzu UV-1650 PC; Shimadzu Europe GmbH, Duisburg, Germany).

### Sequence data processing

Raw sequences were demultiplexed using cutadapt, including removal of primer sequences and low-quality reads [40]. Following, the data was quality filtered, denoised, and chimeric sequences were removed using the DADA2 algorithm and feature table and representative sequences [amplicon sequence variants (ASVs)] were generated [41] within QIIME2 [42]. The ASVs were classified using the SILVA v132 database and the vsearch algorithm [43, 44]. All amplicon libraries were processed separately in QIIME2 and all feature and taxonomy tables were combined to a single phyloseq object in R for further statistical analyses.

### Statistical analyses

Bacterial community analysis was conducted using the package Phyloseq [45] and statistical analysis was performed with R (version 4.3.1) [46] in R studio (version 2023.06.1) [47]. ASVs assigned to “eukaryota”, “archaea”, “chloroplast”, and “mitochondria” were removed from the dataset with the function *subset_taxa*. For beta diversity analysis, the dataset was subjected to cumulative sum scaling and Bray–Curtis dissimilarity matrices were computed. Significant differences were assessed using the function *adonis2* (permutational multivariate analysis of variance—PERMANOVA) from the package VEGAN [48]. To evaluate bacterial alpha diversity the dataset was normalized by random subsampling to 500 reads per sample (Fig. S1A). A total of 15 (out of 704) samples were removed due to low read numbers, a trade-off between sequencing depth and retaining biological replicates (Table S1). The Kruskal–Wallis test was employed to determine significant differences in microbial alpha diversity, based on the Shannon H′ index, species richness, and Faith’s phylogenetic diversity index (PD). PD was calculated using the respective function from the package biomUtilitis [49]. Pairwise comparisons were conducted via Wilcoxon test and *P*-values were corrected with false discovery rate.

General linear models were generated using the *glm* function, followed by a stepwise selection with the function *stepAIC* from the package MASS [50] to identify a minimal fitted model to predict Shannon diversity and observed ASV richness in the roots. Therefore, the dataset was separated and normalized by random subsampling to 4200 reads per sample, whereas three samples were excluded due to a low number of reads (Table S1, Fig. S1B). For each chemical soil parameter used as a predictor variable, an optimal transformation was determined using the *boxcox* function. Distanc-based redundancy analysis (db-RDA) was conducted using the functions *dbrda* and *ordiR2step* implemented in VEGAN [48]. The environmental variables were standardized using the function *decostand* with the “clr” method. For each subplot, five plant samples were obtained, but only one soil core, so the alpha diversity values and the subsampled ASV counts of the five plants were averaged for the regression analysis and the db-RDA, respectively. The sampling site Sweden in the sampling year 2021 had to be excluded from the glm and db-RDA analyses because no soil chemical parameters were collected there.

Core taxa were assessed using the *core_members* function implemented in the package microbiome at various prevalence levels from 0%–100% and a detection level >0.001 on the subsampled dataset [51]. Significant differential abundant genera and phyla were assessed using *DESeq2* incorporated as function *DA.ds2* in the package DAtest and low abundant ASVs with less than 10 reads were trimmed using the function *preDA*. Significant differential abundant genera and phyla were defined by a Benjamini–Hochberg (BH) adjusted *P*-value <.05 and a log2 fold change >0.58 or < − 0.58 corresponding to a fold change of 1.5 [52, 53].

Networks assessing community interactions were created using the package SpiecEasi (version 1.1.2) [54]. The networks were computed for each genotype and field site separately, and to overcome the inflation of zeros, ASVs were filtered per network by a prevalence of 75%. The adjacency matrices were calculated by using Meinshausen–Buhlmann’s neighborhood selection with 50 repetitions, lambda minimum ratio of 0.001, and nlambda of 1000.These lambda settings enabled the calculation of networks with stabilities close to the target stability threshold of 0.05. The network transformation and analysis of network properties were conducted with the package igraph (version 1.3.5) [55]. Global network properties like positive edge percentage, sparsity, and transitivity were calculated along with local network properties for each node, including mean degree, betweenness centrality, closeness centrality, eigenvector centrality, and transitivity. Differences between genotype-specific network parameters were assessed using the Kruskal–Wallis test. Keystone taxa were identified as nodes with an eigenvector centrality value exceeding the empirical 95% quantile [56]. Betweenness centrality is defined by the number of shortest paths going through a node and provides insights into the importance of a taxa based on their role in connecting different parts of the microbial communities. Closeness centrality indicates the proximity of a node to all other nodes, thereby giving insights into its potential to influence them efficiently [57, 58]. Eigenvector centrality takes the connectivity of the associated nodes into account, indicating that a taxon plays a significant role in the overall community by being part of an important subnetwork [59, 60]. Transitivity, also known as clustering coefficient, quantifies the clustering of nodes in a network by measuring the probability that the neighbors of a node are connected and may give indications about niche specialization [57].

## Results

**Figure 1:**
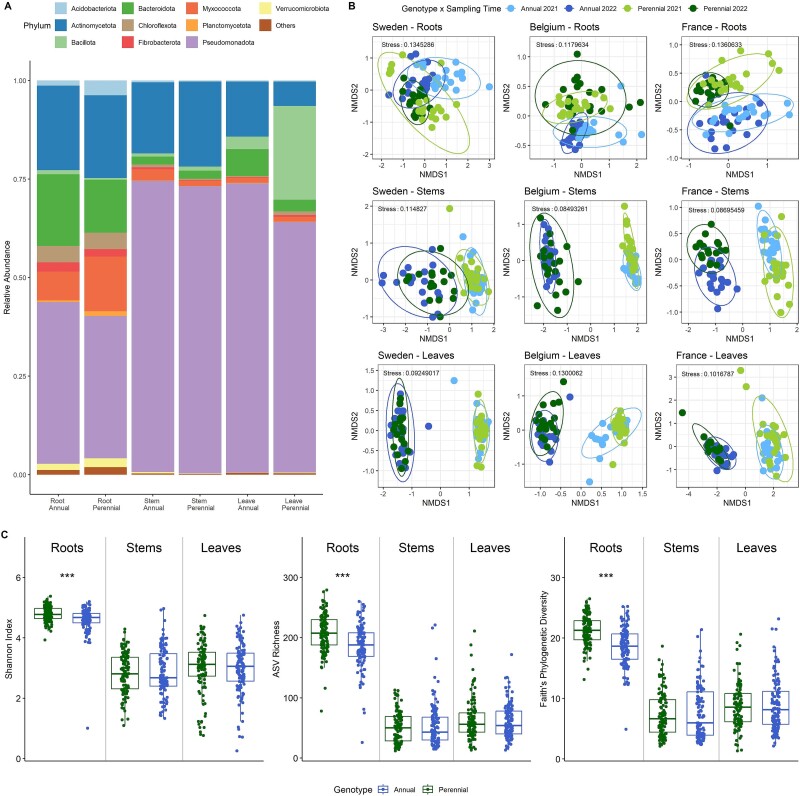
(A) Bacterial taxonomic composition of root, stem, and leaf endophytes from intermediate wheatgrass and annual wheat at phylum level. Samples from three different field sites and two time points (*n* = 114–120 per plant and compartment) were merged. The category “other” was merged from ASVs with a relative abundance below 0.01. (B) Bacterial community composition of intermediate wheatgrass and annual wheat visualized as NMDS plots divided by the main influencing factors field site and plant compartment. The plant genotype is a major source of bacterial community variation in the roots, but to a lesser extent in the aboveground materials (detailed statistics in Table 1). (C) Bacterial alpha diversity in different plant genotypes and compartments depicted as Shannon H′, species richness, and Faith’s phylogenetic diversity. Three sampling sites and two sampling time points were merged (*n* = 114–120). The Kruskal–Wallis test, followed by pairwise comparisons with Wilcoxon testing and “fdr” adjustments, was used to assess significant differences, indicated by asterisks (^**^P < .01 and ^***^P < .001).

In the frame of the NAPERDIV project, 720 plant samples were collected from two plant genotypes (*T. intermedium* L. and *T. aestivum* L.), three endosphere compartments (roots, stems, and leaves), two growth stages (flowering and tillering stage) and three countries (Sweden, Belgium, and France). Previous work showed that the three sites represent different climatic as well as soil conditions (Table S2) [12]. After quality filtering and removal of plant-originating sequences and singletons the final dataset was comprised of 44 704 596 reads, which were classified into 51 832 ASVs and assigned to 48 bacterial phyla. Reads in individual samples ranged from 164 to 2 264 523 with an average number of 6 350.85 ± 148 792.8 reads. The samples were mainly dominated by *Pseudomonadota* (aboveground: 70.9% and roots: 38.5%), followed by *Actinomycetota* (aboveground: 14.8% and roots: 21.2%), and *Bacteroidota* (aboveground: 3.5% and roots: 15.8%; Figs 1A and S2).

### Plant genotype effects on the bacterial composition and diversity

Based on a PERMANOVA analysis, all tested factors, i.e., plant genotype (*R^2^* = 0.5%, *P* = .001), compartment (*R^2^* = 17%, *P* = .001), field site (*R^2^* = 7.5%, *P* = .001), and sampling time point (*R^2^* = 6.8%, *P* = .001), influenced the bacterial community composition. Nonmetric multidimensional scaling (NMDS) plots supported these results as indicated by a clustering mainly in compartment and field site (Fig. S3A–D, Table S6). The root microbiome was mainly influenced by the field site, while the variation in community composition in the aboveground material was mainly explained by the sampling time point (Fig. S4A–C, Table S7).

To further assess the influence of the genotype on the bacterial community structure and how the bacterial composition changed over time, the dataset was split based on the two main influencing factors “compartment” and “field site”. We found that the bacterial community compositions of stems and leaves were mainly influenced by the sampling time point, which resulted in a clear grouping in the NMDS plots at all three sampling sites (Fig. 1B). The genotype explained no (e.g. stems from Sweden), or only little variation in the bacterial community structure (Table 1). The community composition of the roots, on the other hand, showed a clear clustering for the genotype and sampling time point for all three sites. The effect of “genotype” accounted for 7.3% to 12.8% (*P* = .001) of the variation in the root compartment, while the sampling time point explained 4.9% to 12.9% (Table 1). When comparing the three sampling locations, we observed that the samples from Sweden exhibited the lowest “genotype” effect, showing no influence in the aboveground tissues, and a less pronounced effect (*R^2^* = 8.1%, *P* = .001), compared to sampling time point (*R^2^* = 11.7%, *P* = .001), on the root endophytic communities. Similar to the bacterial community composition, a significant difference in alpha diversity between the annual and perennial genotypes was only observed in the root compartments. The diversity and richness in stems (Shannon: *P* < .0005; Faith PD: *P* < .0005; observed: *P* < .0005) and leaves (Shannon: *P* < .0005; Faith PD: *P* < .0005; observed: *P* < .0005) were notably lower than those in the roots, but with similar levels across both genotypes (Fig. 1C, Fig. S7). However, the perennial roots exhibited higher diversity (Shannon: *P* < .0005; Faith PD: *P* < .0005) and richness (*P* < .0005) compared to their annual counterparts.

**Table 1:** Effects of plant genotype and sampling time on bacterial community composition of different compartments and sites assessed with PERMANOVA.

	Plant genotype		Sampling time		Plant genotype × sampling time	
	R^2^ (%)	Pr (>F)	R^2^ (%)	Pr (>F)	R^2^ (%)	Pr (>F)
France—roots	12.8	0.001	12.9	0.001	4.3	0.001
Belgium—roots	7.3	0.001	4.9	0.001	8.1	0.001
Sweden—roots	8.1	0.001	11.7	0.001	3.1	0.001
France—stems	4.3	0.001	21.5	0.001	7.2	0.001
Belgium—stems	1.8	0.051	31.1	0.001	1.6	0.067
Sweden—stems	1.3	0.245	13.2	0.001	1.6	0.102
France—leaves	2.9	0.004	19.4	0.001	2.8	0.004
Belgium—leaves	2.5	0.004	22.1	0.001	2.3	0.009
Sweden—leaves	1.7	0.052	27.2	0.001	1.8	0.045

### Effect of soil parameters on the root-endophytic microbiome

To determine the influence of genotype and chemical soil properties on differences in bacterial diversity observed in the roots, generalized linear models were applied. This was followed by stepwise selection from both directions to identify a minimal set of predictor variables included in the best fitted models (Fig. S8–S10). From the eight variables tested, four significant ones remained in the final models (Table 2, Fig. S5). The adjusted McFadden’s R^2^ was computed for the final models to assess their adequacy, resulting in a value of 0.506 for the Shannon diversity and 0.501 for ASV richness. It was shown that the ratio of carbon:nitrogen (C:N) was the strongest predictor for Shannon diversity and ASV richness. In addition, “respiration” and “water content” exhibited a significant influence on both indices. Furthermore, an increase in “microbial C:N” and “dehydrogenase activity” resulted in an increase in Shannon diversity and richness, respectively. Interestingly, the factor “plant genotype” was not included in either of the final models. Furthermore, plant available potassium and phosphorus were excluded from the most adequate models.

**Table 2:** Effects of environmental variables on root Shannon H′ index and observed richness explained by generalized linear models and on the root bacterial community composition based on db-RDA ANOVA.

Parameter	Shannon diversity		ASV richness		Beta diversity		
	F value	Pr (>F)	F value	Pr (>F)	F value	Pr (>F)	R^2^ adj.
C:N	16.7162	0.0003	15.8215	0.0004	3.35	0.001	4.83
Respiration	8.9843	0.0054	6.4275	0.0169	2.38	0.007	2.79
Water content	5.1286	0.0309	6.7556	0.0145	-	-	-
Microbial C:N	8.0094	0.0082	-	-	6.27	0.001	13.43
DHA	-	-	9.3124	0.0048	5.74	0.001	10.87
Phosphorus	-	-	0.6588	0.4236	-	-	-
Plant genotype	-	-	-	-	4.35	0.001	7.21
Potassium	-	-	-	-	-	-	

To assess the influence of plant genotype and environmental variables on the root bacterial communities a distance-based redundancy analysis was applied. A stepwise selection from both directions was applied and the final model had an adjusted R^2^ of 41.9% (*P* = .0001). It was shown that microbial C:N, DHA, plant genotype, C:N, and respiration could significantly explain the variance in the bacterial community composition, while water content, phosphorus and potassium were excluded from the best fitted model (Table 2, Fig. S6).

### Network analysis of the root microbiome

**Figure 2:**
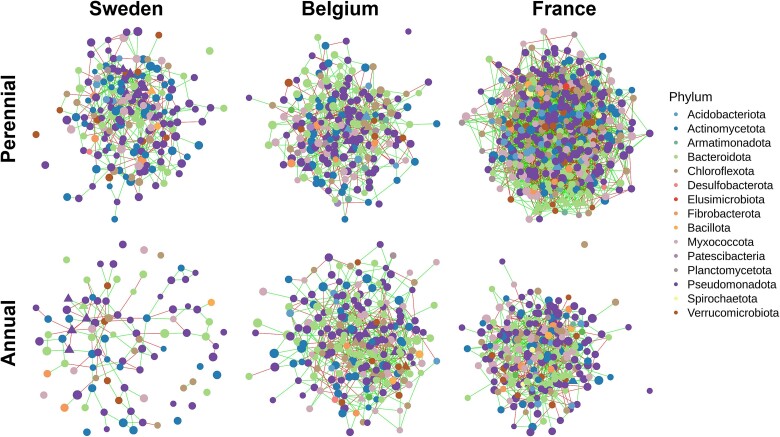
Co-occurrence networks showing the structure of the root microbiome from different plant genotypes and field sites inferred by SPIEC-EASI. The two sampling time points were merged and only ASVs with a prevalence >0.75 were included (*n* = 34–40). Node color and size correspond to the taxonomy on phylum level and clr-transformed abundance, respectively. Edge colors represent positive (green) and negative (red) associations between ASVs. Keynote taxa are represented as triangles.

Networks were calculated to investigate the taxonomic relationships of bacterial communities for the different plant genotypes and field sites. In order to assess the stability of the root microbiomes, the sampling time points were merged and only ASVs with a prevalence of 75% were kept for network analyses. All networks exhibited a similar density (0.0221–0.0255), but the topology differed distinctively between the plant genotypes, but also the field sites (Table 3, Fig. 2, Fig. S11). The network structures resembled the sampling gradient from North to South, with networks from Sweden exhibiting the lowest number of nodes and edges, while those from France had the highest values. A higher number of edges per node was found in the networks of intermediate wheatgrass in France and Sweden, while the networks of annual wheat exhibited a higher percentage of positive edges in France and Belgium. All three networks of intermediate wheatgrass depicted significantly higher values for the local network parameter closeness centrality and the ones from Sweden and France had a significantly higher average number of neighbors and a greater number of keystone taxa. A substantial number (18 out of 42) of the keystone taxa in the perennial networks belong to the family of *Chitinophagales* and *Rhizobiales*, while the keystone taxa in the annual networks were dominated (10 out of 30) by *Chitinophagales* and *Burkholderiales* (Table S3). Betweenness centrality was higher in the annual wheat networks from Sweden and France, however this was not statistically significant. The annual networks of Sweden and Belgium showed significantly higher eigenvector centralities. Transitivity was only significantly higher in the network of annual wheat from France, while no difference was observed at the other field sites.

**Table 3:** Topological parameters of root microbiome networks of different plant genotypes and field sites. Two sampling time points were merged and only ASVs with a prevalence >0.75 were included in the network construction.

Prevalence filter: 0.75%									
Field site	Sweden			Belgium			France		
Genotype	Perennial	Annual	*P*-value	Perennial	Annual	*P*-value	Perennial	Annual	*P*-value
Stability	0.0499	0.0497		0.0495	0.0495		0.05	0.0495	
Nodes	194	97		218	222		395	248	
Edges	457	120		524	548		1781	679	
Positive edges	294	75		322	360		1080	428	
Positive edges in %	64.332604	62.5		61.45038168	65.693431		60.6400898	63.03387	
Negative edges	163	45		202	188		701	251	
Edges/Nodes	2.3556701	1.237113		2.403669725	2.4684685		4.50886076	2.737903	
Pos/Neg edges	1.803681	1.666667		1.594059406	1.9148936		1.54065621	1.705179	
Sparsity	0.0243	0.0255		0.0221	0.0222		0.0228	0.0221	
Max degree	16	9		13	16		18	15	
Mean degree	4.73	2.47	**2.56E-13**	4.81	4.94	0.6267	9.02	5.48	**< 2.2e-16**
Betweenness centrality	0.028	0.044	0.9982	0.027	0.025	0.9531	0.0132	0.0204	0.1347
Closeness centrality	11.17	5.51	**< 2.2e-16**	13.94	11.68	**1.18E-09**	28.93	15.13	**< 2.2e-16**
Eigenvector centrality	0.052	0.07	**0.0006**	0.036	0.122	**< 2.2e-16**	0.094	0.119	0.1296
Number of keystone taxa	10	5		11	12		20	13	
Transitivity	0.094	0.12	0.121	0.086	0.08	0.3365	0.0742	0.0802	**0.03521**

Significant results are highlighted in bold.

To further test the hypothesis, that perennial wheatgrass root communities changed less over the two sampling time points than the annual wheat communities, core microbiome analyses were conducted. Thereby we assumed that a higher number of ASVs was shared between the two sampling time points. The dataset was again split according to field site and genotype. Neither intermediate wheatgrass nor annual wheat had a core at a prevalence of 100%, indicating that no ASV was found in every sample of either plant genotype. This observation was consistent even when the samples were analysed per field site (Table S4). The shared core between the two sampling time points was comprised of a higher number of ASVs in the intermediate wheatgrass than the core of annual wheat at most prevalence levels in Sweden and Belgium. At the field site in France, the annual wheat had slightly higher numbers of shared ASVs at prevalence levels of 0.375–0.625. Similarly, the percentage of ASVs in the shared core compared to the respective unique cores was, at most prevalence levels and field sites, higher in the intermediate wheatgrass (mean: 50%) than in annual wheat (mean: 42%). The most pronounced decrease in the number of ASVs in the shared core was for all sample types from the prevalence level 0–0.125, suggesting a variable fraction of rare bacteria only present in a few samples. Interestingly, while both genotypes from the site Sweden exhibited high numbers of ASVs at low prevalence levels, they had the smallest core at the higher prevalence levels.

**Figure 3:**
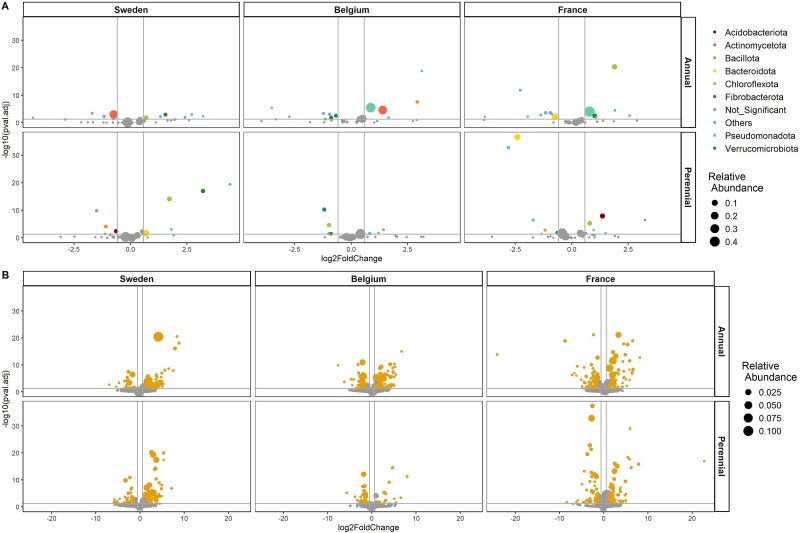
(A) Differentially abundant phyla between the two sampling time points (left: 2021, right: 2022). Phyla that are significantly different abundant (BH adjusted *P* < .05) and a log2 fold change >0.58 or < 0.58 are represented by colored points, while phyla below the threshold are gray and labeled as “Not_Significant”. The group “others” represent phyla with a relative abundance below “1%”. The point size corresponds to the mean relative abundance. (B) Differential abundant genera between the two sampling time points (left: 2021, right: 2022). Genera that are significantly different abundant (BH adjusted *P* < .05) and a log2 fold change >0.58 or < 0.58 are represented by orange points, while genera below the threshold are gray. The point size corresponds to the mean relative abundance.

To assess which bacterial phyla were most affected between the two sampling time points, differential abundance analyses were conducted at phyla and genera level. Phyla and genera were considered significantly differential abundant if they exhibited an adjusted *P*-value <.05 and a log2 fold change >0.58 or < −0.58. At all three sites, the intermediate wheatgrass root microbiome showed a lower number of significantly different abundant phyla, compared to annual wheat (Table S5). The difference was most pronounced in Belgium (perennial: 25.8%; annual: 39.3%) and the least dominant in Sweden (perennial: 24.3%; annual: 28.9%; Table S5, Fig. 3A). This pattern was further observed in the abundance of the changed phyla. The relative abundance of the largely unchanged phyla was consistently higher in intermediate wheatgrass (Sweden: 77.1%; Belgium: 92.4%; France: 77.5%) compared to the annual counterpart (Sweden: 68.6%; Belgium: 34.2%; France: 34.8%; Fig. 3A). The observed differences in the relative abundance in annual wheat were due to the abundance change in the most dominant phyla (e.g. *Pseudomonadota* and *Actinomycetota*), which were not significantly different in intermediate wheatgrass. On genus level the differences between intermediate wheatgrass and annual wheat were less pronounced (Fig. 3B). The number of significantly changed genera was lower for intermediate wheatgrass compared to annual wheat at the field site Belgium (perennial: 9%; annual: 23.3%). For the field sites Sweden (perennial: 16.6%; annual: 15.3%) and France (perennial: 19.6%; annual: 19.9%) the number of significantly differential abundant genera was similar. A similar pattern was observed for the abundance of the largely unchanged genera, with major differences between the plant genotypes at the field site Belgium (perennial: 85.6%; annual: 60.8%), but only minor differences at the field sites France (perennial: 55.5%; annual: 59.6%) and Sweden (perennial: 52.1%; annual: 49.5%).

## Discussion

The results obtained in this study provide fundamental insights into the dynamics of bacterial communities associated with perennial wheatgrass and its annual counterpart wheat. Higher diversity of the bacterial community and differences to annual wheat in terms of composition were mainly shown for the belowground compartment of intermediate wheatgrass. Furthermore, the root microbiome of intermediate wheatgrass collected from the three different sampling sites showed signatures of a more stable and connected microbial network structure.

### Chemical and biological soil parameters influence the bacterial community structure of root endophytes

Perennial crops are important for different ecosystem services, partially due to their commonly extensive root systems [61]. Ecosystem services provided by them include positive effects on soil health [7, 62, 63]. Soil serves as an important reservoir of diverse microbial communities, from which plants can specifically select bacteria that can inhabit the plant endosphere [64, 65]. We identified several soil chemical and biological parameters influencing the root bacterial diversity and community composition. Interestingly, the plant genotype partially explained the variance in the community composition but was not included in the best fitted models for alpha diversity. This means that the plant genotype does not provide additional information to the models explaining diversity beyond what is already accounted for by the soil chemical (e.g. soil water content) and biological (e.g. soil microbial C:N, DHA) parameters. However, other variables, such as the C:N or the water content were included in the models and showed negative correlations with diversity and richness. It was previously shown that the C:N ratio and soil water content are, among others, influencing not only the soil microorganisms but also the structure of plant endophytic microbiome [66]. Furthermore, Bak *et al.* [67] observed that intermediate wheatgrass exhibits, unlike other deep-rooting plant species, high abundances of the N-cycling genes *nirK* and *nifH* in the root environments which indicates that N fixation contributes to plant N supply. The fixed N can subsequently be utilized by root-associated bacteria through N-rich plant exudates [67]. Therefore, it was speculated that the high relative abundance of N fixers has the potential to increase microbial biomass (microbial C) by the decrease in the C:N ratio of plant exudates [67]. In line with this, we were able to show that an increase in the soil microbial C:N ratio was correlated with an increase in diversity in roots.

In particular, soil biological parameters related to microorganisms, such as respiration, microbial C:N ratio, and DHA explained part of the alpha diversity. Interestingly, respiration showed a negative effect on both alpha diversity indices that were assessed. It was previously discussed that biodiversity and community functioning are closely interconnected [68]. It was shown in controlled experiments that once diversity reaches a certain saturation level of community functioning, further increases in diversity do not have a significant impact on community functioning [69]. This is especially relevant for common functions like respiration. Last, DHA can serve as an indicator of microbial activity and is frequently employed for microbial redox systems [70]. Most studies focused on the assessment of DHA in association with soil contamination and it was shown that there is a limited correlation with soil microbial diversity [71, 72]. In our study, an increase in DHA and the microbial C:N ratio in the soil was correlated with higher alpha diversity in the roots. In previous studies comparing the rhizosphere or soil of annual wheat and perennial wheatgrass, the latter was shown to accumulate higher microbial biomass and support higher bacterial activity [16, 73]. Hence, we suggest that the higher bacterial diversity and richness observed in intermediate wheatgrass roots are influenced by factors beyond plant genotype, such as soil quality. Intermediate wheatgrass promotes a more active soil microbial community with higher microbial biomass [73], therefore we speculate that the perennial lifecycle of the dense rooted intermediate wheatgrass and/or differences in the management create an environment that facilitates higher bacterial diversity. This, in turn, could result in a larger reservoir of microorganisms from which the plant can select, creating a beneficial feedback loop.

### The plant genotype mainly influenced the root microbiome composition and diversity and to a lesser extent the aboveground compartments

We found that primarily the plant compartment, the sampling location and the time influenced the bacterial community composition more than the genotype of the cereal. A similar pattern was previously observed for sugarcane [74]. While we observed that the root microbiome was mainly influenced by the sampling site, the aboveground structures were mostly affected by the sampling time point. Furthermore, the host genotype effect was more prominent in the belowground compartment. Similar results were observed for lucerne [75] and maize [76] where belowground tissues, but not the leaves, of different plant genotypes were shown to harbor distinct bacterial communities. To disentangle the host genotype effect, the dataset was separated based on the two primary influencing factors, plant compartment, and sampling site. A clear trend was observed for the bacterial composition of root and aboveground compartments at all three sampling sites. While the root microbiomes clustered by genotype and sampling time point, the aboveground microbiome was mainly influenced by the sampling time point. In addition, we observed a higher bacterial diversity and richness in the roots of intermediate wheatgrass compared to annual wheat, whereas no significant differences were noted in the stems and leaves between the two plant species. Given the management practices used for intermediate wheatgrass cultivation, it is important to note that wheatgrass is typically cut a few centimeters above the ground during harvesting [77, 78]. Therefore, the aboveground material has to regrow each year at the onset of the vegetation period, resulting in a comparable growth process to annual plants. However, the extensive root system of intermediate wheatgrass is less affected by the harvesting process and can continue to develop over the years. In contrast, in annual wheat, the root system (and aboveground material) must develop from a single seed and completely re-establish itself every growing season. While the intermediate wheatgrass root microbiome can continuously be shaped by the host, plants in annual management are more prone to environmental disturbances due to soil operations, including priority effects. Therefore, we suggest that the root systems of intermediate wheatgrass are the key compartment that differentiates perennial plants from annual ones, while the aboveground structures share characteristics of annual plants.

It was previously shown by Bertola *et al.* [16] that one-year old perennial wheat had a comparable rhizosphere microbial structure to annual wheat. However, upon analyzing four-year old perennial wheat stands, the authors found that they resembled the 11-year old plants of intermediate wheatgrass and were distinct from the one-year old wheat (perennial and annual) stands. This pattern highlights the conserved ecological niche of perennial roots, enabling the development of a distinct root microbiome. The authors further hypothesize that the root microbiome development becomes saturated, probably due to a rather stable surrounding [16]. We compared bacterial structures of intermediate wheatgrass and annual wheat over two time points and found more connected networks in intermediate wheatgrass with lower values for betweenness. Higher connectivity, characterized by higher mean degree and closeness centrality values, has been linked with higher system stability due to redundancy. However, it has also been suggested that a high level of connectivity may render systems more susceptible to cascade effects [79]. Nodes with high betweenness centrality are often termed as “gatekeepers” and networks with low betweenness centrality values may indicate higher stability [79–81]. Intermediate wheatgrass networks showed more competition, indicated by a low ratio of positive to negative edges, which is generally associated with ecological stability [82, 83]. Moreover, we observed a higher number of core ASVs and less significant changes in the relative abundance on the phylum level in intermediate wheatgrass. These patterns were detected at all three field sites. This was expected, as annual wheat cultivation is subjected to different environmental disturbances, such as soil tillage. Previous work showed that soil bacterial networks, as well as cross-domain networks, including fungi and protists, follow a gradient of land-use intensity. The networks of the permanent grasslands exhibited higher levels of connectivity and complexity than those under continuous cropping and temporary grasslands, resembling intermediate wheatgrass cultivation, fell within a gradient between the two [10]. It is important to note that comparisons between endophytic communities in intermediate wheatgrass and annual wheat must go beyond discussions of differences between plant genotypes. The management varies between the two plant types (e.g. tillage) and it is hardly possible to distinguish between these two factors. Targeted sampling strategies, e.g. by comparing several perennial and annual plant types or investigation of land-use gradients, will be necessary in the future to disentangle the differences between effects from plant genotypes and the cropping system. Yet, the management is an integral part of the cultivation of perennial grain crops and should be considered beside the factor plant genotype.

In conclusion, we observed consistent findings across the three sampling sites showing compartment-specific bacterial communities resembling the host plant lifestyle (perennial vs. annual). Importantly, the root-endophytic microbiome of intermediate wheatgrass showed higher diversity and more connected communities. At the same study sites, intermediate wheatgrass cultivation was found to improve the diversity, abundance, and biomass of earthworms and to favor a nematode community structure that is characteristic for an undisturbed system with a more diverse food web [12, 84]. Similar patterns across macro- and microorganism scales emphasize the potential of intermediate wheatgrass cultivation for fostering sustainability in agriculture by increasing biodiversity.

## Supplementary Material

Supplementary_Material_ycae165

Supplementary_Tables_ycae165

## Data Availability

Raw sequencing data for each sample used in this study was deposited at the European Nucleotide Archive (ENA) in the FASTQ format and is available under the Bioproject accession number PRJEB74910.
