# Supplementary material for: The microbiome of a perennial cereal differs from annual winter wheat only in the root endosphere
Source: ISME Commun. 2024 Dec 23;5(1):ycae165. doi: 10.1093/ismeco/ycae165 (PMC11812607; doi:10.1093/ismeco/ycae165)
Supplement: Supplementary_Material_ycae165 [file supplementary_material_ycae165.pdf]

Supplementary Material for:

The microbiome of a perennial cereal differs from its annual counterpart only in the rhizosphere

Kristina Michl[1], Makoto Kanasugi[2,3], Alena Förster[4], Regina Wuggenig[1], Sulemana Issifu[5], Katarzyna Hrynkiewicz[3], Christoph Emmerling[4], Christophe David[6], Benjamin Dumont[7], Linda-Maria Dimitrova Mårtensson[8], Frank Rasche[5,9], Gabriele Berg[1,10,11], and Tomislav Cernava[1,12]

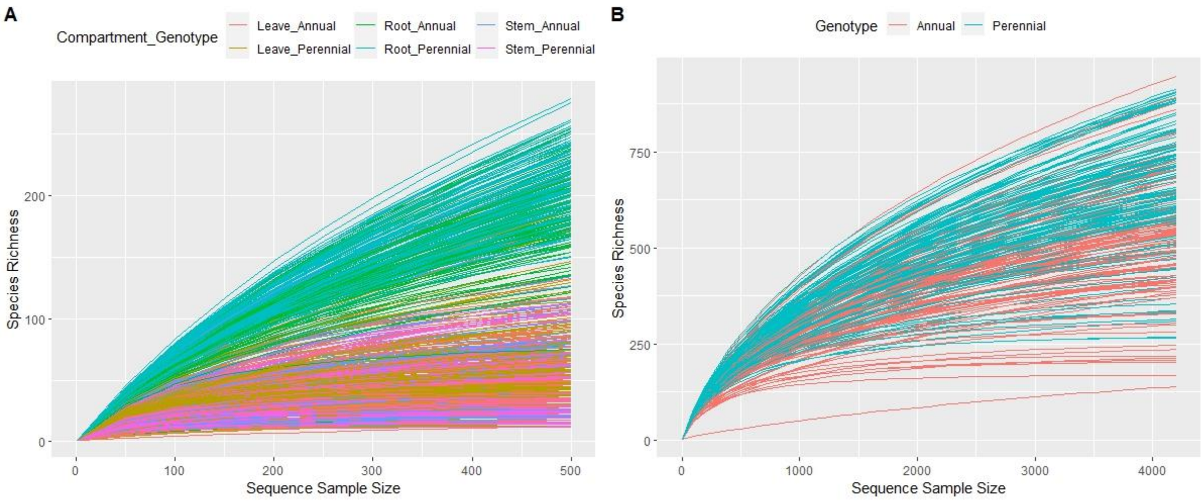

Figure S1. *Rarefaction curves of 16S rRNA gene amplicons. (A) All samples were rarefied to 500 reads per sample. (B) Only root samples were rarefied to 4200 reads per sample.*

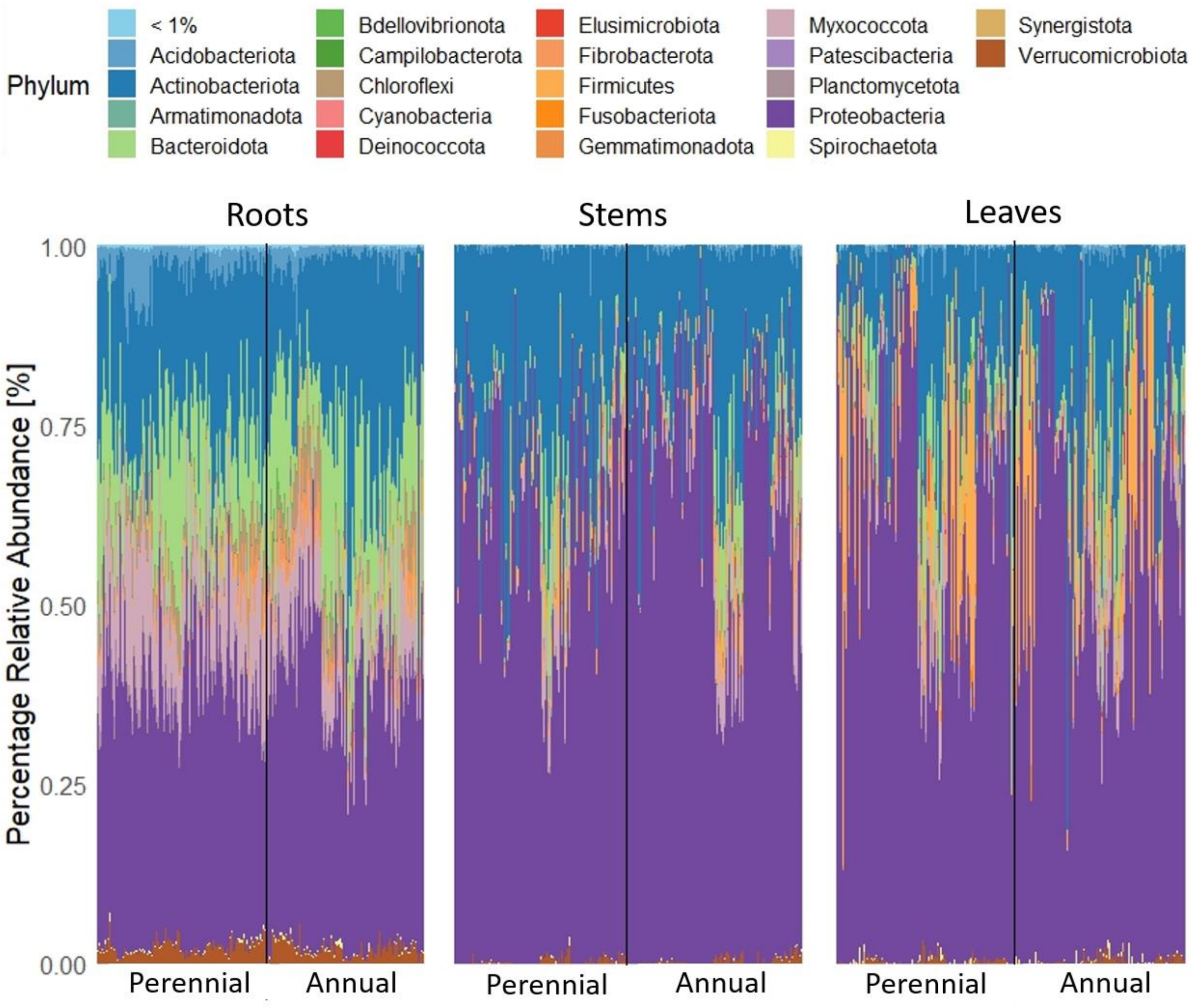

Figure S2. *Bacterial taxonomic composition of root, stem, and leaf endophytes from intermediate wheatgrass and annual wheat at phylum level. Samples were collected from three different field sites and two timepoints (n=140-120 per plant and compartment). The category "<1%" was merged from ASVs with a relative abundance below 1%.*

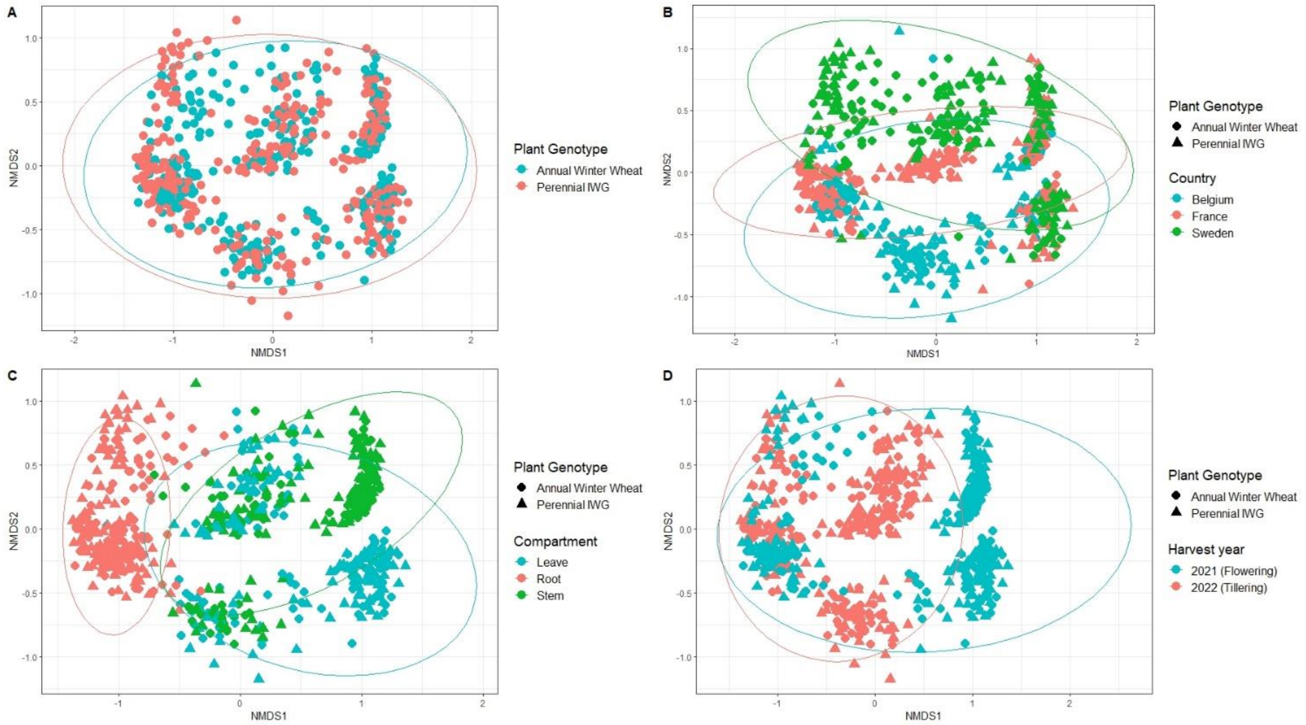

Figure S3. *Bacterial community composition of all samples (n=704) depicted as NMDS plots. The clustering was only to a lesser extent influenced by (A) plant genotype, but mainly by (B) field site, (C) compartment, and (D) sampling time.*

Table S6. *Effects of different parameters on bacterial community composition of all samples combined (n=704) assessed with PERMANOVA.*

	R^2	Pr (>F)
Genotype	0.005	0.001
Compartment	0.075	0.001
Sampling Site	0.17	0.001
Sampling Time	0.068	0.001
Genotype*Country	0.007	0.001
Genotype*Compartment	0.012	0.001
Country*Compartment	0.038	0.001
Genotype*Sampling Time	0.002	0.004
Sampling Site*Sampling Time	0.035	0.001
Compartment*Sampling Time	0.061	0.001
Genotype*Sampling Site*Compartment	0.013	0.001
Genotype* Sampling Site*Sampling Time	0.005	0.001
Genotype*Compartment*Sampling Time	0.004	0.001
Sampling Site*Compartment*Sampling Time	0.024	0.001
Genotype*Sampling Site*Compartment*Sampling Time	0.01	0.001
Residuals	0.472	

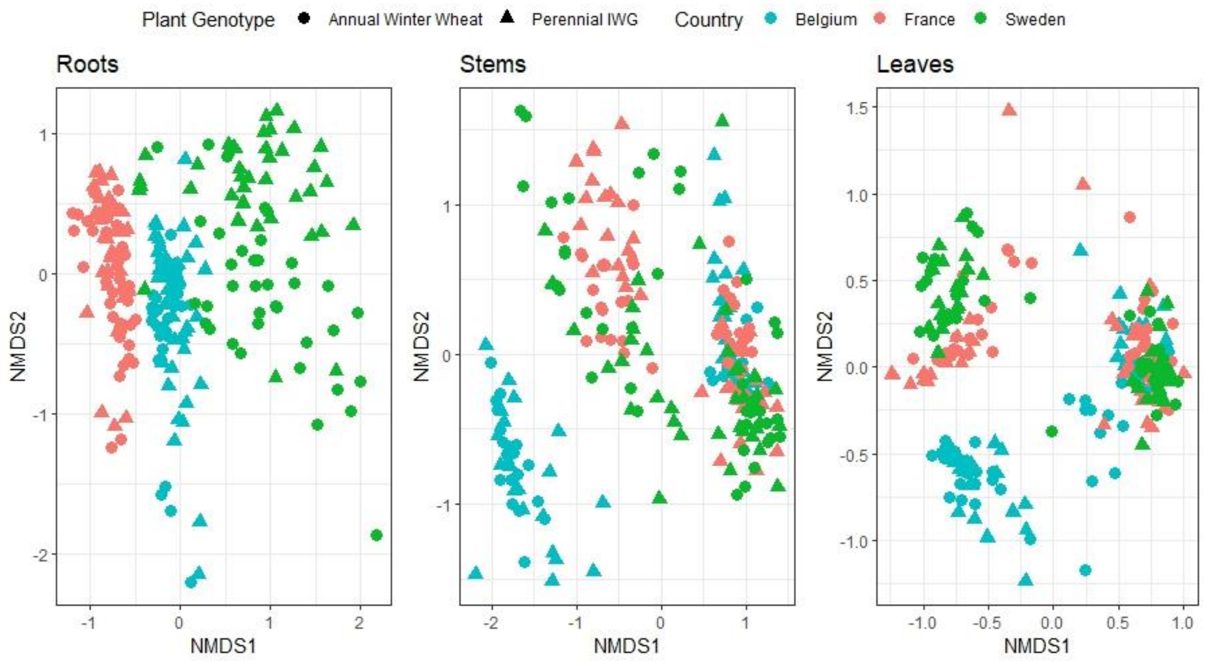

Figure S4. *Bacterial community composition separately analyzed for (A) roots, (B) stems, and (C) leaves depicted as NMDS plots.*

Table S7. *Effect of plant genotype, sampling site, and sampling time on bacterial community composition assessed with PERMANOVAs.*

	Roots		Stems		Leaves	
	R^2	Pr (>F)	R^2	Pr (>F)	R^2	Pr (>F)
Genotype	0.042	0.001	0.009	0.001	0.007	0.002
Sampling Time	0.028	0.001	0.189	0.001	0.215	0.001
Sampling Site	0.25	0.001	0.099	0.001	0.072	0.001
Genotype*Sampling Time	0.01	0.001	0.008	0.001	0.006	0.023
Genotype*Sampling Site	0.039	0.001	0.016	0.001	0.015	0.001
Sampling Time*Sampling Site	0.04	0.001	0.085	0.001	0.072	0.001
Genotype*Sampling Time*Sampling Site	0.019	0.001	0.024	0.001	0.013	0.002

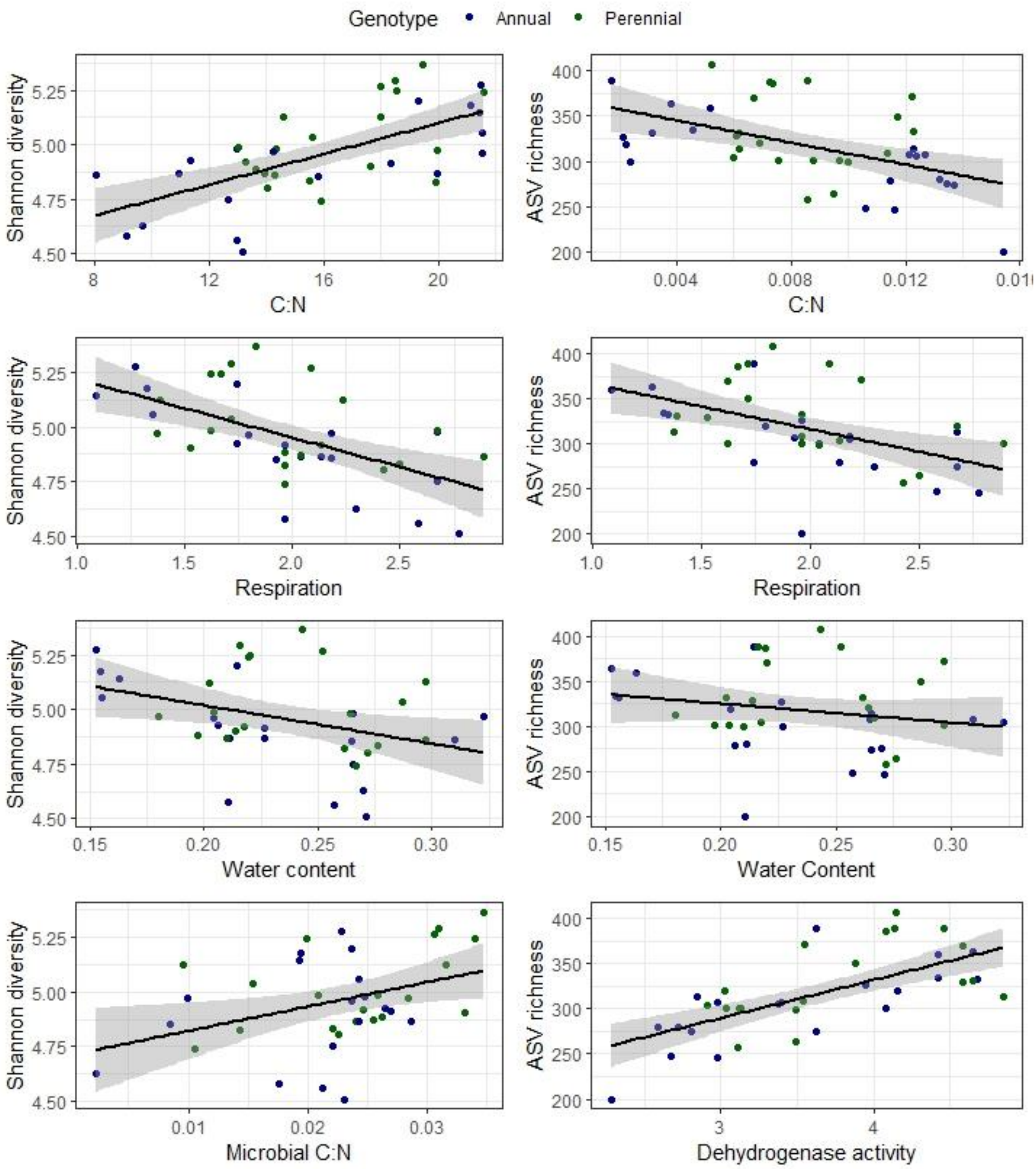

Figure S5. *Bacterial alpha diversity detected in roots, depicted as Shannon diversity or ASV richness, in relation to soil chemical and biological parameters assessed via generalized linear models.*

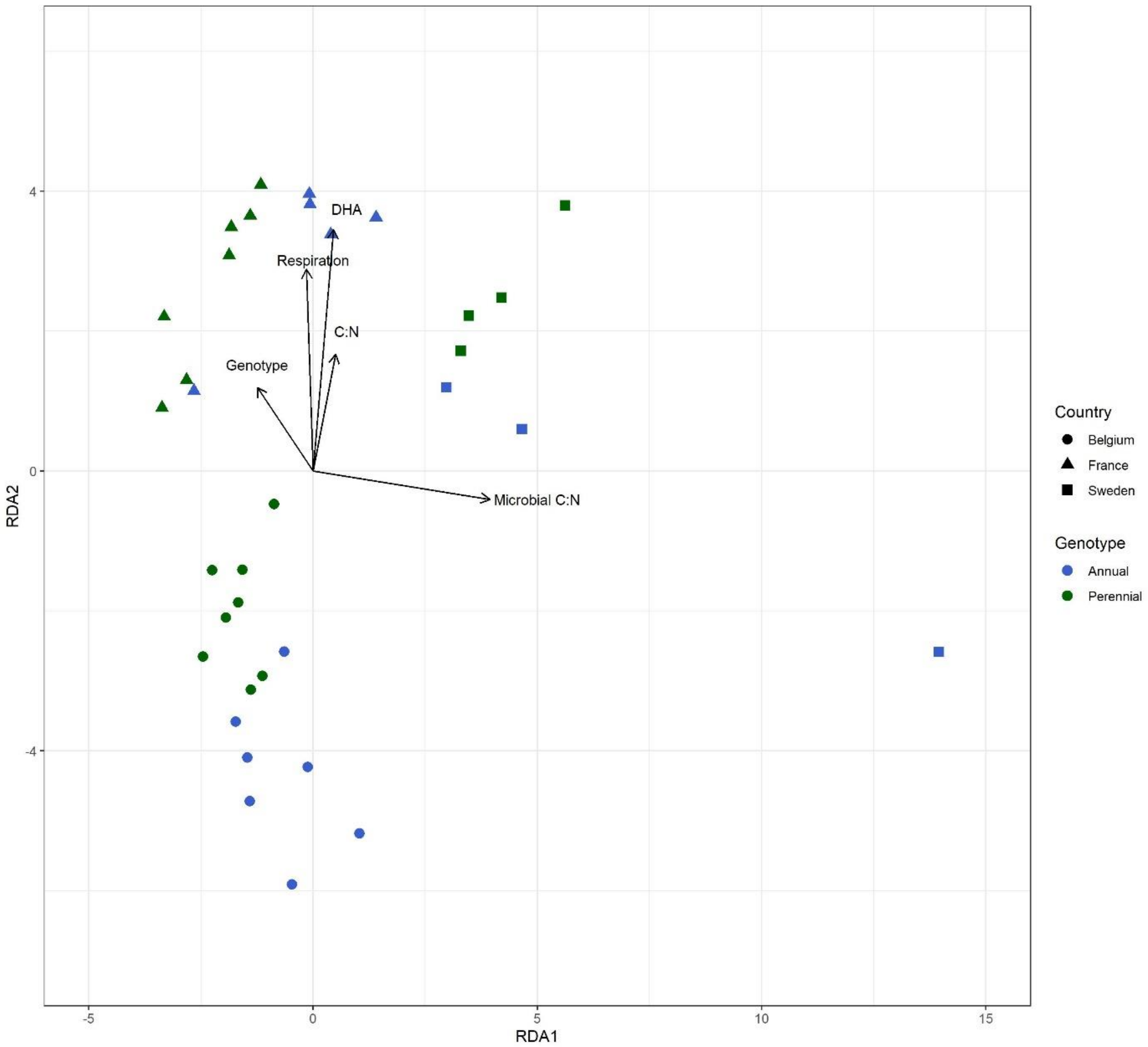

Figure S6. *Redundancy analysis of root bacterial community composition. Arrows denote the direction in which the gradient of the environmental variable is greatest, while the length of the arrow reflects the strength of the correlation between the environmental variables and the bacterial community composition. Only significant variables, determined through stepwise regression with selection from both sides are presented.*

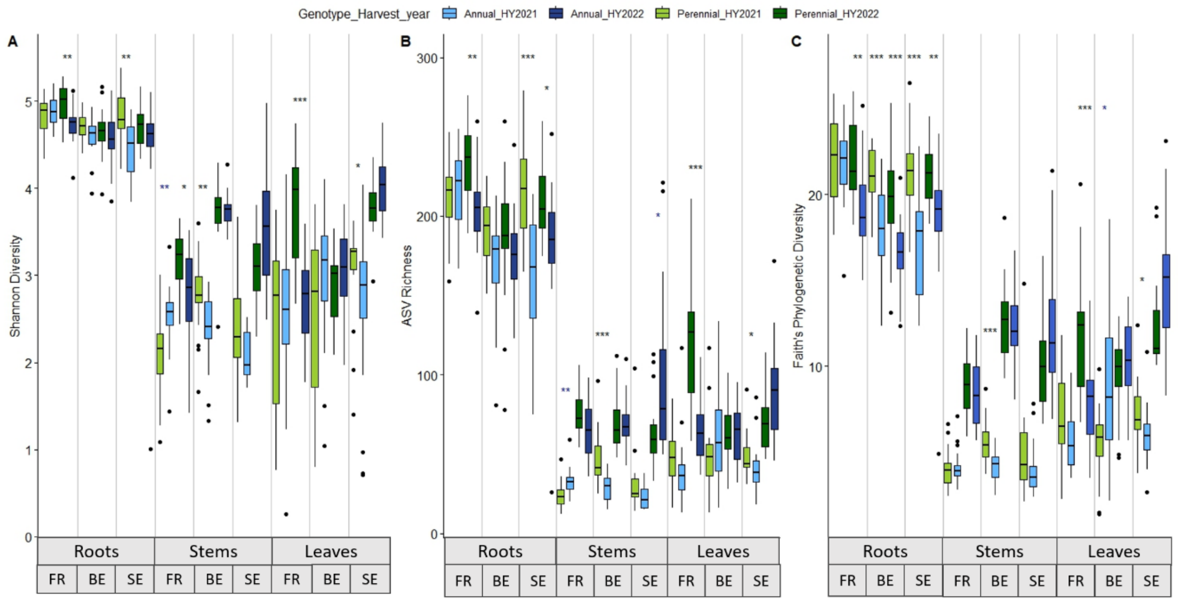

Figure S7. *Alpha diversity separately assessed for plant genotype, compartment, sampling site, and sampling time as (A) Shannon H', (B) Species Richness, and (C) Faith's Phylogenetic Distance. The dataset was rarefied to 500 reads per sample. Statistically significant differences were assessed using the Kruskal-Wallis test, followed by pairwise comparisons via Wilcoxon test and "false discovery rate" adjustment.*

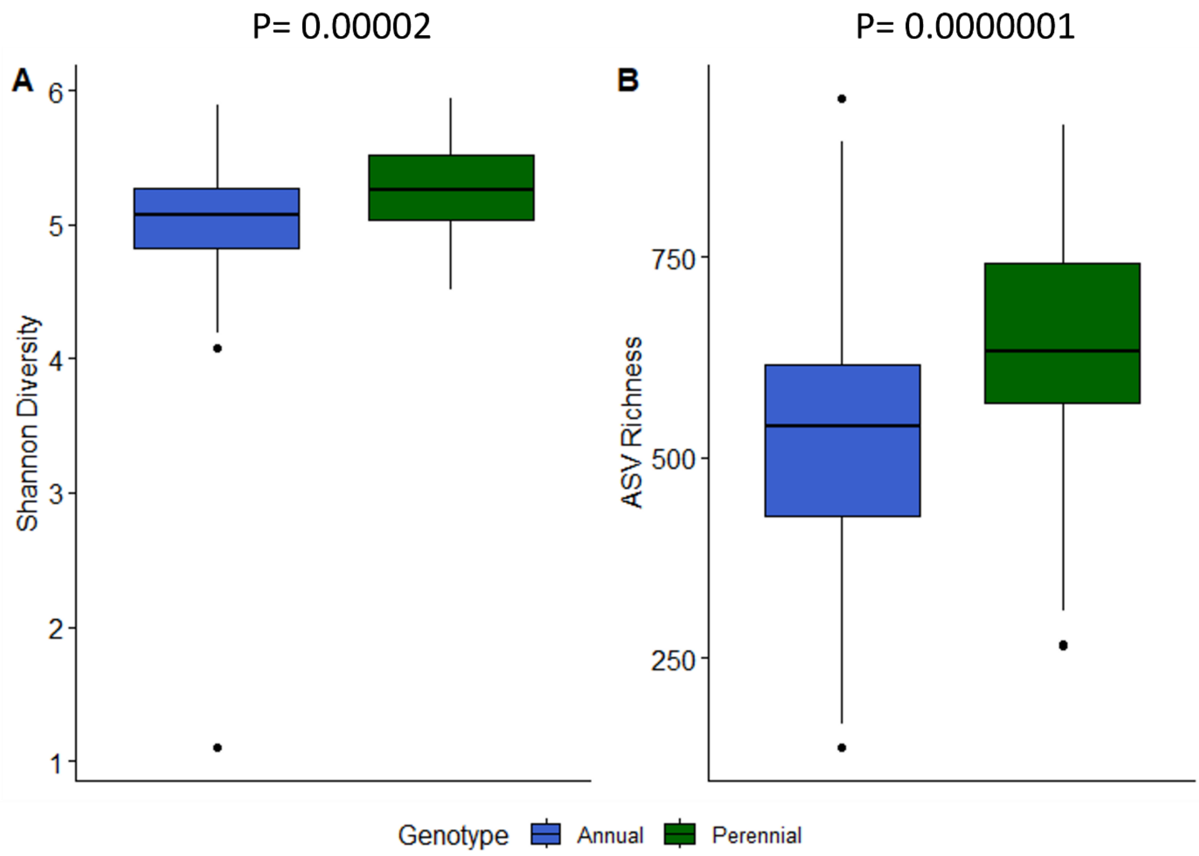

Figure S8. *Alpha diversity assessment of intermediate wheatgrass and annual wheat root microbiomes as (A) Shannon H' diversity and (B) ASV richness. The dataset was normalized to 4200 reads per sample. Statistically significant differences were computed by Kruskal-Wallis test.*

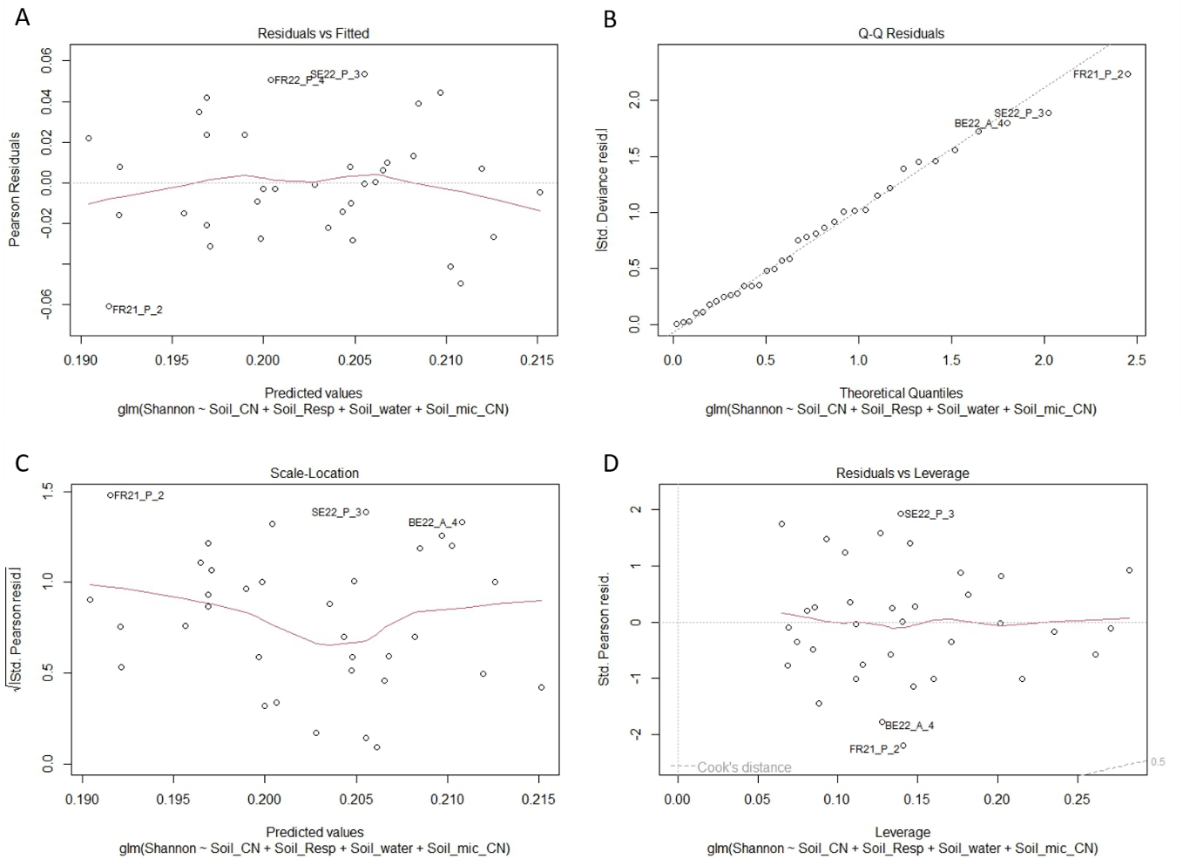

Figure S9. *Graphs depicting the residuals of the best fitted generalized linear model predicting Shannon diversity.*

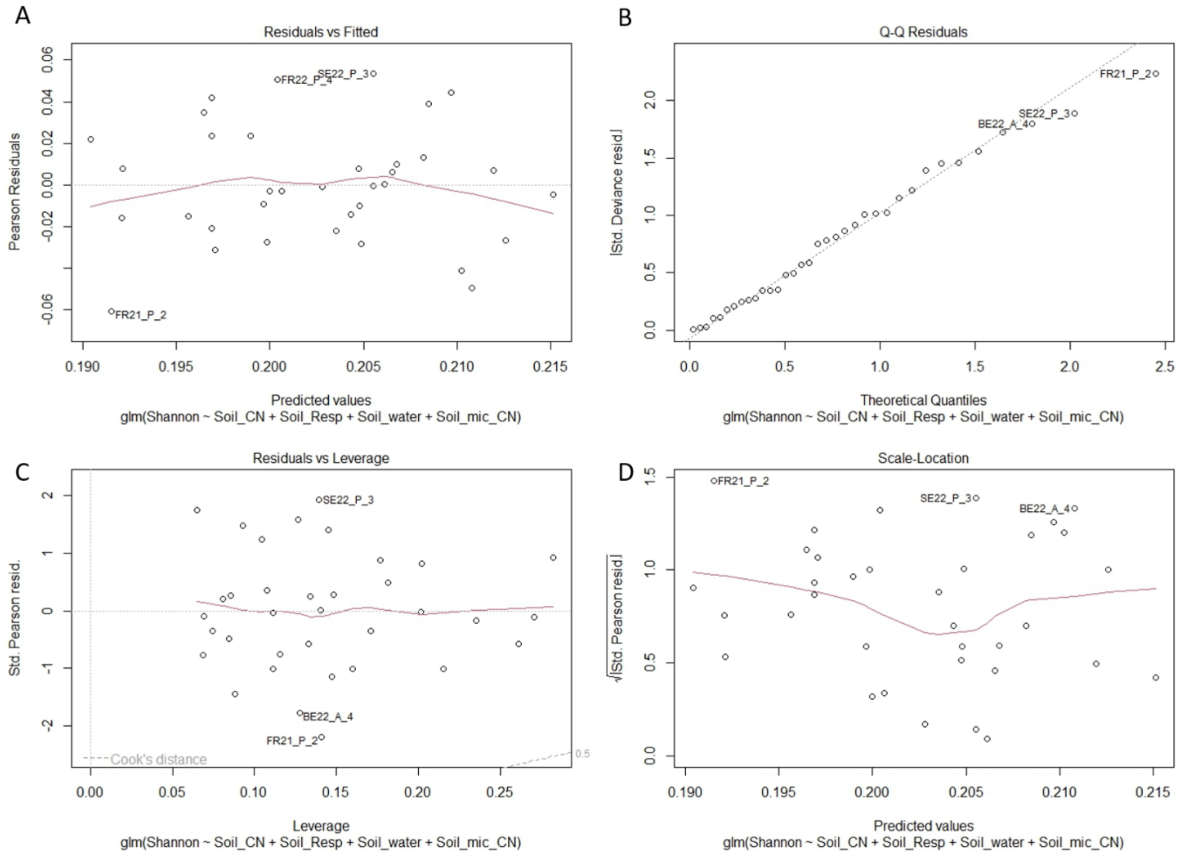

Figure S10. *Graphs depicting the residuals of the best fitted generalized linear model predicting ASV richness.*

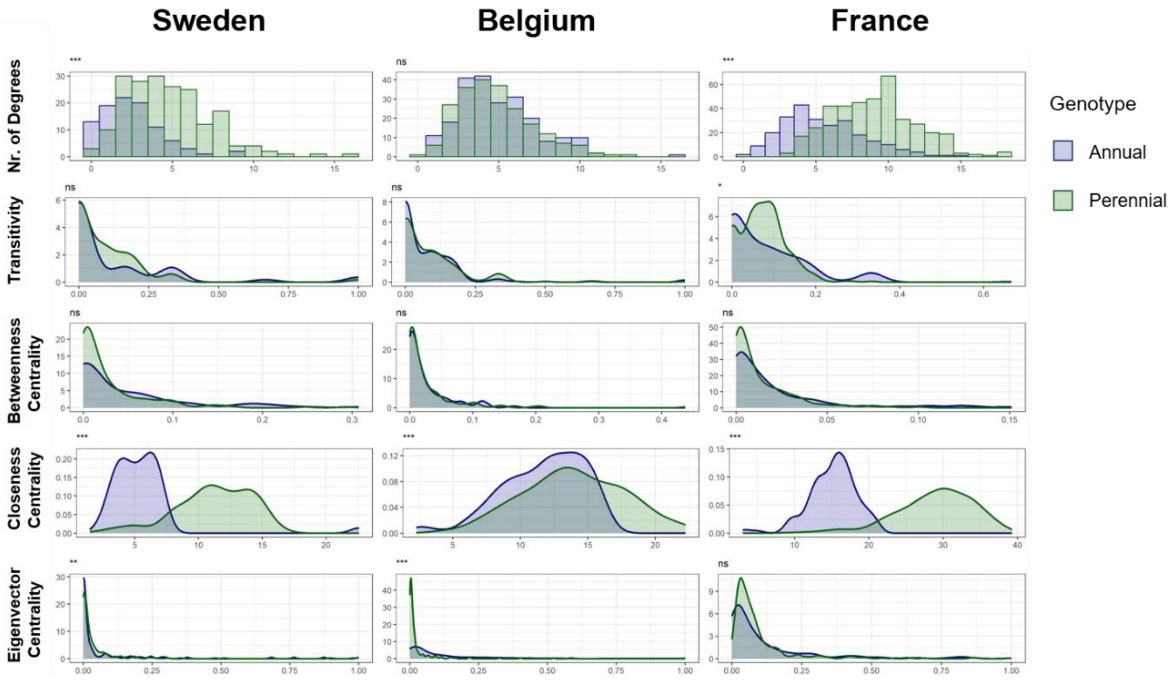

Figure S11. *Distribution of local network parameters from the field sites Sweden, Belgium, and France (left to right). Difference between genotype-specific network parameters were assessed using the Kruskal-Wallis test.*
